# Physical activity and sedentary behaviour typologies of 10-11 year olds - Response to Saunders and Colleagues

**DOI:** 10.1186/1479-5868-8-49

**Published:** 2011-05-25

**Authors:** Russell Jago, Kenneth R Fox, Angie S Page, Rowan Brockman, Janice L Thompson

**Affiliations:** 1 Centre for Exercise, Nutrition & Health Sciences, School for Policy Studies, University of Bristol, Bristol, UK

## 

Dear Editor,

We read with interest the recent letter by Saunders and colleagues [1] in relation to our paper entitled "Physical activity and sedentary behaviour typologies of 10-11 year olds" [2] and we welcome the opportunity to respond to the comments that have been raised in their letter. Firstly, they question why we opted to use self-reported activity data to generate behavioural profiles when objective accelerometer data were available. Secondly, they asked us to explain why the differences in the accelerometer-determined physical activity levels were relatively small when the self-reported differences were far larger.

Regarding their first point, the self-reported physical activity and screen-viewing data were used to create profiles of the types of behaviour in which the participants engaged. The questions used focussed on frequency of attendance of sport clubs, playing with friends near the home, playing with friends in the garden as well as time spent screen-viewing. We made no assumptions about the actual intensity of the physical activity in which the participants engaged, and thus did not claim that if a participant reported attending an after-school club for 1-2 days per week that this led to an extra amount of physical activity. This decision was taken because we are aware that a large proportion of time that children spend participating in clubs or sports is not moderate to vigorous in intensity [3]. More importantly, however, the instrument was not intended to provide an indication of overall levels of physical activity. Rather the questions were designed to provide information on the *types *of behaviours in which the participants engaged. These data are needed as although accelerometers can provide detailed information on the intensity of physical activity and the time of day at which it occurred, they cannot provide information about what a person was doing when they were physically active. While accelerometer data can provide information about whether or not a child meets physical activity guidelines, the data cannot solely inform interventions as there is no information about the activities in which the child engages. Context of activity is required to guide and target strategies for promoting activity in children. We therefore used self-reported data to identify children who reported engaging in similar behaviours with the intention being that this information might then be used to design targeted interventions for children with similar physical activity and screen-viewing profiles.

Regarding their second point, Saunders and colleagues are correct to point out that the differences between the clusters in terms of self-reported participation in physical activity and screen-time were far larger than the differences when analysed via accelerometer. However, it is important to be clear that the outcomes were the accelerometer-derived variables and not the self-reported physical activity participation or screen-viewing time. We acknowledge that some of the screen-viewing estimates may not be plausible but they provide a good reflection of the perceived screen-viewing behaviours and patterning in relation to other children which, as concluded in the paper, can inform the design of interventions.

In summary, we believe the analyses that we performed and the interpretation of those analyses are correct. We agree with Saunders and colleagues that accelerometers provide more accurate representations of volumes and intensities of physical activity than self-reported activity participation but, in this paper, the activity participation data were used to provide context on what the children were doing when accruing accelerometer-derived physical activity. We therefore believe that our objective could not have been met by the singular analysis of accelerometer data as suggested by Saunders and colleagues, which we feel answers an interesting, but ultimately different question.

Sincerely,

Russ Jago, Ken Fox, Angie Page, Rowan Brockman and Janice Thompson - University of Bristol, UK

## References

[B1] Saunders TJ Prince SA Tremblay MS Clustering of children's activity behaviour: the use of self-report versus direct measures Int J Behav Nutr Phys Act 2011 8 48 10.1186/1479-5868-8-48 21612580 PMC3119190

[B2] Jago R Fox KR Page AS Brockman R Thompson JL Physical activity and sedentary behaviour typologies of 10-11 year olds Int J Behav Nutr Phys Act 2010 7 59 10.1186/1479-5868-7-59 20663226 PMC2918527

[B3] Trost SG Rosenkranz RR Dzewaltowski D Physical activity levels among children attending after-school programs Med Sci Sports Exerc 2008 40 622 629 10.1249/MSS.0b013e318161eaa5 18317385

